# High Serum Uric Acid Is Associated with Tubular Damage and Kidney Inflammation in Patients with Type 2 Diabetes

**DOI:** 10.1155/2019/6025804

**Published:** 2019-04-11

**Authors:** Naiara S. Guarda, Yãnaí S. Bollick, José Antonio M. de Carvalho, Melissa O. Premaor, Fabio V. Comim, Rafael N. Moresco

**Affiliations:** ^1^Laboratory of Clinical Biochemistry, Department of Clinical and Toxicological Analysis, Federal University of Santa Maria, Santa Maria, RS, Brazil; ^2^University Hospital, Federal University of Santa Maria, Santa Maria, RS, Brazil; ^3^Department of Clinical Medicine, Federal University of Santa Maria, Santa Maria, RS, Brazil

## Abstract

**Background:**

Uric acid presents different roles in an organism. High serum uric acid concentrations may induce inflammatory pathways and promote kidney damage through different mechanisms. Therefore, this study investigated the association among high serum uric acid concentrations, renal tubular damage, and renal inflammation assessed via estimation of urinary kidney injury molecule-1 (KIM-1) and inflammatory cytokines in patients with type 2 diabetes (T2D).

**Methods:**

Urinary concentrations of KIM-1, IL-1, IL-6, IL-10, and TNF-alpha, as well as other biochemical parameters, were assessed in 125 patients with T2D who were grouped into two groups based on the serum uric acid levels (<6.0 mg/dL and ≥6.0 mg/dL). Patients were also stratified according to the tertiles of serum uric acid concentrations.

**Results:**

Urinary KIM-1, IL-1, IL-6, and TNF-alpha were higher in patients with serum uric acid concentrations ≥ 6.0 mg/dL. However, the differences between the groups were not statistically significant when the urinary values of KIM-1 and cytokines were normalized by the urinary creatinine concentration. Serum uric acid concentrations were significantly associated with urinary KIM-1 (values normalized by urinary creatinine concentration) and urinary TNF-alpha (absolute values and values normalized by urinary creatinine concentration), independent of the body mass index (BMI) and estimated glomerular filtration rate (eGFR).

**Conclusions:**

High serum uric acid concentrations were associated with high urinary KIM-1 levels accompanied by the increase of urinary proinflammatory cytokines in patients with T2D. However, normalization of urinary markers by urine creatinine concentration seems to influence the profile of the results.

## 1. Introduction

Hyperuricemia has emerged as a risk factor for the development of different conditions such as diabetes and cardiovascular and kidney diseases [1–4]. The association between diabetes and hyperuricemia has been extensively investigated, as such the role of uric acid in the physiopathology of diabetic kidney disease (DKD). However, the exact mechanism by which uric acid acts at the renal level is not fully understood. It is not known whether uric acid has a direct nephrotoxic effect or if it promotes some conditions leading to kidney damage. Uric acid induces hyalinosis and thickening of preglomerular arterioles, and it promotes endothelial dysfunction, glomerular hypertrophy, and activation of the renin-angiotensin system [5]. All these factors may lead to the development of kidney disease.

Increased serum uric acid is an independent risk factor for the onset and progression of microalbuminuria both in patients with diabetes and in those without diabetes [5, 6]. Uric acid has the potential to cause damage not only at the glomerular level but also at the tubular level [7]. It is known that renal uric acid reabsorption occurs mainly in the proximal tubules [8]. Likewise, its generation occurs in tubular cells suggesting that uric acid can act directly on this portion of the kidney [9]. Tubular cells are important targets for the onset and progression of kidney damage. Hence, biomarkers of tubular damage can contribute substantially to improve the early detection of renal injury [10]. The kidney injury molecule-1 (KIM-1), a new marker of tubular injury, is a type I cell membrane glycoprotein expressed at low levels in healthy kidneys [11]. It has been shown that normoalbuminuric patients with type 2 diabetes (T2D) presented increased urinary levels of KIM-1 [12].

Inflammation is another pathway which plays a key role in the pathophysiology of kidney damage [13, 14]. The tubulointerstitial lesion is characterized by infiltrates of inflammatory cells with tubular atrophy and consequent interstitial fibrosis [10]. In the renal environment, inflammatory molecules such as cytokines play an important role in the development of DKD [15]. In this context, some cytokines have been highlighted as interleukin 1 (IL-1), interleukin 6 (IL-6), and tumor necrosis factor-alpha (TNF-alpha) [16]. Interestingly, uric acid is a proinflammatory factor since it can induce the expression of IL-6 and TNF-alpha through the factor nuclear kappa B (NF-*κ*B) signaling pathway [17, 18]. In the kidneys, these cytokines promote changes in renal and hemodynamic structures and cellular necrosis, as well as changes in the permeability of the glomerular endothelium [15, 19, 20].

Although evidence shows the involvement of hyperuricemia and inflammation in the pathophysiology of diabetes and its chronic complications, it is still not fully understood whether elevated serum uric acid concentrations are capable of promoting increased tubular damage and renal inflammation in patients with T2D. Therefore, the aim of the present study was to investigate the association among high serum uric acid concentrations, renal tubular damage, and renal inflammation assessed via estimation of urinary KIM-1 and inflammatory cytokines in patients with T2D.

## 2. Materials and Methods

### 2.1. Study Population

Overall, 196 patients with T2D enrolled at the University Hospital of Santa Maria (Rio Grande do Sul, Brazil) were examined in this study. Exclusion criteria included urinary tract diseases, previous renal disease other than DKD, infectious diseases, liver diseases, acute or chronic inflammatory diseases, pregnancy, medical history of kidney transplantation or malignancy, and use of nephrotoxic drugs and anti-inflammatory and/or immunosupressive drugs. Finally, 125 patients were eligible for the study (85 females and 40 males). The study protocol was approved by the Institutional Ethics Committee (12303113.0.0000.5346), and written informed consent was obtained from all patients. The patients were grouped into 2 groups based on the serum uric acid levels: <6.0 mg/dL (*n* = 90) and ≥6.0 mg/dL (*n* = 35). This classification was assumed because the study population consisted mainly of female patients (68%) and because 6.0 mg/dL is considered the upper limit of the reference range for female individuals. Furthermore, patients were also stratified according to the tertiles of serum uric acid concentrations. Clinical characteristics and medical histories of the patients were collected via a clinical and epidemiological assessment questionnaire or from the hospital's medical register. We followed in part the methods described by Stein et al. [21]. However, in the present study, a larger number of patients and new laboratory parameters were investigated.

### 2.2. Sample Collection and Laboratory Assays

Blood samples were collected from all patients, after an overnight fast period of at least 8 h, by venous puncture technique into Vacutainer® tubes (BD Diagnostics, Plymouth, UK) containing EDTA, sodium fluoride plus EDTA, or no anticoagulants. The blood samples were centrifuged at 2500 ×*g* for 15 min. Fasting glucose was measured using plasma, while serum was used to assess uric acid, total cholesterol, HDL cholesterol, LDL cholesterol, and triglycerides. These measurements were performed using standard methods on the Dimension RxL Max® automated analyzer (Siemens Healthcare Diagnostics Inc., Malvern, Pennsylvania, USA). The EDTA containing whole blood samples were used to measure glycated hemoglobin (HbA_1c_) by use of a chromatographic method on the D-10® analyzer (Bio-Rad, California, USA). First-morning urine samples were obtained from the patients and centrifuged at 1000 ×*g* for 5 min. The supernatants were used to measure albumin, creatinine, KIM-1, IL-1, IL-6, IL-10, and TNF-alpha. Urinary albumin was quantified by use of an immunoturbidimetric assay on the Dimension RxL Max® automated analyzer, and the results were expressed as the albumin/creatinine ratio (ACR) as a tool to match the levels of albumin in accordance with the concentration of urine [22]. Urinary KIM-1, IL-1, IL-6, IL-10, and TNF-alpha were measured using commercial ELISA kits (R&D Systems Inc., Minneapolis, Minnesota, USA). All tests were performed according to the instructions recommended by the manufacturer. The estimated glomerular filtration rate (eGFR) was calculated using the creatinine equation obtained from the Chronic Kidney Disease Epidemiology Collaboration (CKD-EPI) [23].

### 2.3. Statistical Analysis

The variables were tested for normality using the D'Agostino-Pearson test. The parametric variables are presented as mean ± standard deviation (SD), and the nonparametric variables are presented as the median and interquartile range (IQR). Statistical differences between the groups were analyzed using Student's *t* test or the Mann-Whitney test. The categorical data are expressed as percentages, and differences between the groups were compared using the Chi-square test. Additionally, multiple regression analysis was performed to investigate the influence of some variables on urinary KIM-1 levels. Two-tailed *P* values < 0.05 were considered statistically significant. All results were analyzed using GraphPad Prism® version 6.00 (GraphPad Software, La Jolla, California, USA) and Statistica® version 9.1 (StatSoft Inc., Tulsa, Oklahoma, USA).

## 3. Results

The baseline characteristics of the participants included in the study are shown in Table 1. No differences in age, the proportion of smokers, and the proportion of patients with T2D were observed between the groups. However, significant differences were observed concerning gender, BMI, and proportion of hypertensive patients. Plasma fasting glucose, blood HbA_1c_, serum LDL cholesterol, and eGFR levels were not statistically different between the groups. However, serum total cholesterol levels were higher, and HDL cholesterol levels were lower in the group with uric acid ≥ 6.0 mg/dL, when compared with the group with serum uric acid < 6.0 mg/dL.

**Figure 1:**
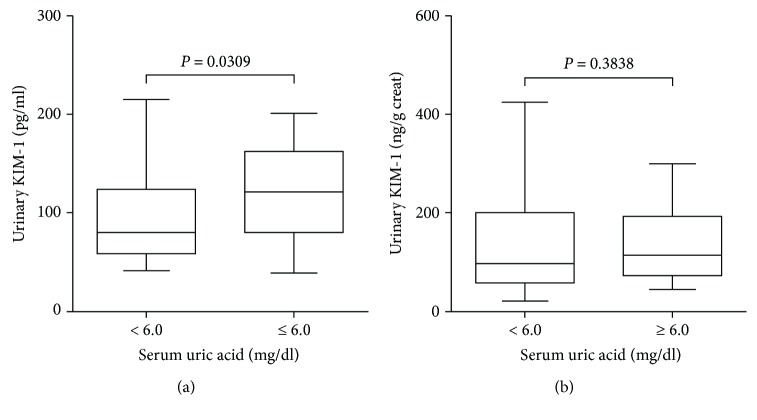
Box-and-whisker plots showing the urinary values of KIM-1 reported as (a) absolute values and (b) values normalized by urinary creatinine concentration. Subjects (*n* = 125) were stratified based on serum uric acid levels < 6.0 mg/dL and ≥6.0 mg/dL. The box contained 50% of all values (from the 25^th^ to 75^th^ percentile) and was divided by the horizontal bar representing the median value (50^th^ percentile).

**Figure 2:**
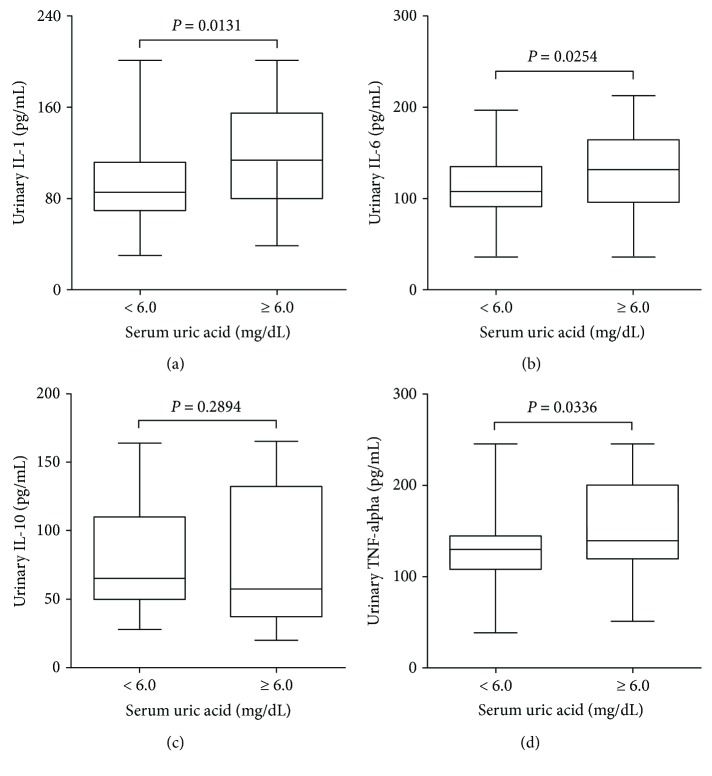
Box-and-whisker plots are showing the urinary concentrations of IL-1 (a), IL-6 (b), IL-10 (c), and TNF-alpha (d) reported as absolute values. Subjects (*n* = 125) were stratified based on serum uric acid levels < 6.0 mg/dL and ≥6.0 mg/dL. The box contained 50% of all values (from the 25^th^ to 75^th^ percentile) and was divided by the horizontal bar representing the median value (50^th^ percentile).

**Figure 3:**
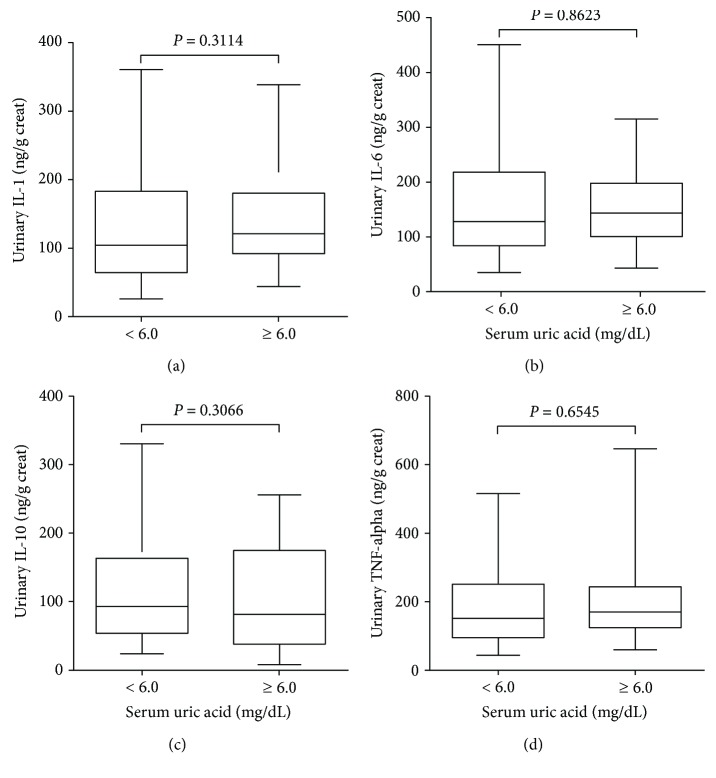
Box-and-whisker plots are showing the urinary concentrations of IL-1 (a), IL-6 (b), IL-10 (c), and TNF-alpha (d) reported as values normalized by urinary creatinine concentration. Subjects (*n* = 125) were stratified based on serum uric acid levels < 6.0 mg/dL and ≥6.0 mg/dL. The box contained 50% of all values (from the 25^th^ to 75^th^ percentile) and was divided by the horizontal bar representing the median value (50^th^ percentile).

The urinary KIM-1 levels were significantly higher in patients with high serum uric acid when compared with those with low serum uric acid (122 (80–162) versus 80 (58–124) pg/mL, *P* = 0.0309), as shown in Figure 1(a). However, the difference between the groups was not significant when the urinary values of KIM-1 were normalized by the urinary creatinine concentration (Figure 1(b)). The ACR levels observed in the patients did not differ statistically between the groups (Table 1). Furthermore, urinary concentrations of IL-1, IL-6, and TNF-alpha were significantly higher in patients with high serum uric acid when compared with those with low serum uric acid, as shown in Figure 2. Although a decreasing trend in the urinary IL-10 in the group of patients with high serum uric acid was observed, the difference between the groups was not statistically significant. Interestingly, the differences were not statistically significant when urinary cytokine concentrations were normalized by the urinary creatinine concentration (Figure 3).

Additionally, the patients were stratified according to the tertiles of serum uric acid concentrations (1^st^ tertile: 2.7–4.5 mg/dL, 2^nd^ tertile: 4.6–5.7 mg/dL, and 3^rd^ tertile: 5.8–9.0 mg/dL). Based on this stratification, there were differences between the groups only for KIM-1 (absolute values and normalized by urinary creatinine concentration), as shown in Table 2.

Although differences in urinary cytokines were not statistically significant, there was a trend toward elevation of IL-1, IL-6, and TNF-alpha in the third tertile. The multiple regression analysis was conducted using two models that included urinary KIM-1 and urinary cytokines, as well as other variables. Serum uric acid concentrations were significantly associated with urinary KIM-1 (values normalized by urinary creatinine concentration) and urinary TNF-alpha (absolute values and values normalized by urinary creatinine concentration), independent of the BMI and eGFR (Table 3).

## 4. Discussion

The associations among serum uric acid levels, tubular damage, and renal inflammation were investigated in the present study. Interestingly, we observed the cooccurrence of high serum uric acid with high urinary KIM-1, IL-1, IL-6, and TNF-alpha in patients with T2D. However, these differences were no longer significant when the values were corrected by the urinary creatinine concentration. This was an intriguing finding since there is no definite consensus on the need for normalization of urinary KIM-1 and cytokines by the concentration of creatinine in the urine.

Adjustment of results by urinary creatinine is usually performed to compensate for variations in urine concentrations, but the urinary excretion of creatinine can vary for different reasons. Most evidence seems to support the use of tubular biomarkers such as KIM-1 expressed as values corrected for urinary creatinine [24–27]. However, some studies expressed the results of KIM-1 only as values adjusted by urinary creatinine and not as absolute values. Thus, it is not possible to conclude on the influence of the adjustment of the KIM-1 results by urinary creatinine on the performance of the marker in these clinical conditions. Conti et al. [28] investigated the effect of urinary creatinine on urinary cystatin as a model of a tubular marker, and urinary cystatin was superior to the urinary cystatin/creatinine ratio to evaluate the extent of renal tubular damage. Therefore, it was recommended to avoid the adjustment of tubular markers to urinary creatinine, especially in patients with acute or even moderate chronic renal failure. Interestingly, a study from our group reported that urinary KIM-1 had a higher ability to discriminate incipient diabetic kidney disease than the urinary KIM-1/creatinine ratio [29]. In contrast, another study showed that both urinary cystatin C and KIM-1 concentrations were positively correlated with urinary creatinine, indicating a rationale for urinary creatinine adjustment [30].

Some studies have investigated the role of uric acid in causing kidney damage [3–6, 31]. The uric acid at high levels has the potential to induce renal injury through different mechanisms such as renal vasoconstriction mediated by endothelial dysfunction, inflammation, activation of the renin-angiotensin system, and afferent renal arteriolopathy [31–35]. Uric acid may promote the phenotypic transition of renal cells even at physiological concentrations [7]. Likewise, high concentrations of uric acid may cause tubulointerstitial fibrosis [7]. Furthermore, a recent study from our group [21] reported the cooccurrence of high serum uric acid with high urinary 8-hydroxydeoxyguanosine (8-OHdG), a marker of nucleoside oxidation, in patients with T2D. We speculate that serum uric acid at high concentrations combined with other prooxidant changes observed in diabetes appears to potentiate the formation of 8-OHdG in T2D patients [21].

In the present study, we investigated the influence of serum uric acid on tubular damage in patients with T2D and observed that it occurred most strongly in patients with T2D with higher serum uric acid concentrations. Interestingly, a previous study reported the occurrence of high concentrations of urinary KIM-1 in adolescents with hyperuricemia compared to healthy adolescents [36]. Another finding of the present study was that similar ACR levels were found in both groups of patients. It is known that tubular damage may precede and even occur independently of glomerular dysfunction. For this reason, the assessment of markers of early tubular renal damage such as KIM-1 becomes a valuable tool [37–39]. Following tubular damage, the injured kidney cells have deranged expression and secretion of KIM-1, and it is a potential marker for monitoring the degree of tubular epithelial cell injury [39]. Although the exact mechanism of the released soluble KIM-1 remains unknown, it has been suggested that KIM-1 can participate in the injury process since it plays an important role in the proliferation and regeneration processes in proximal tubules [11, 39, 40].

Inflammation has an important role in the pathophysiology of diabetes mellitus, and it contributes significantly to the development of renal disease [41, 42]. Interestingly, uric acid appears to play a key role both in inflammation and in kidney damage [32, 43]. Furthermore, uric acid may promote renal inflammation via the NF-*κ*B signaling pathway [44]. The production of interleukin-1*β* resulting from the induction of the NLRP3 pathway in renal mesangial cells through the action of uric acid appears to be another important mechanism for the development of kidney damage [45]. We had previously reported an increase of urinary IL-6 combined with a decrease in urinary IL-10 in patients with DKD [46]. However, in the present study, we investigated the influence of serum uric acid on renal inflammation in patients with T2D and observed that it occurred most evidently in patients with T2D with higher serum uric acid concentrations. Thus, we speculate that serum uric acid at high concentrations, when combined with other metabolic and prooxidant changes observed in diabetes, seems to enhance the proinflammatory cytokines involving IL-1, IL-6, and TNF-alpha in T2D patients. However, differences were not statistically significant when urinary cytokine concentrations were normalized by the urinary creatinine concentration. Thus, it is important to reflect on the need to correct the results of urinary cytokines by the concentration of creatinine in the urine, similar to what happens with tubular markers such as KIM-1. It is well established that proinflammatory cytokines may promote renal effects through several pathways. IL-1 and IL-6 can promote hypercellularity and interstitial fibrosis in the renal tubules [47, 48], while TNF-alpha has cytotoxic action on mesangial and epithelial glomerular cells [15, 49].

Unfortunately, this study has some limitations. Firstly, the number of patients enrolled was relatively small. For this reason, some of the associations reported in the present study were not very strong, which may have been influenced by this relatively small number of investigated patients. Secondly, the potential contribution of systemic cytokine concentrations to their urinary levels was not known since serum levels of cytokines were not measured. Thirdly, first-morning urine samples were used to quantify urinary biomarkers rather than timed urine specimens, which would allow a more accurate estimate of the excretion rate. Furthermore, it was not possible to investigate in more depth the effects of the use of certain drugs that may affect serum uric acid on urinary markers measured in this study.

## 5. Conclusion

High concentrations of serum uric acid were associated with tubular damage accompanied by the increase of urinary proinflammatory cytokines in patients with T2D. However, normalization of urinary markers by urine creatinine concentration seems to influence the profile of the results. Therefore, a standardization for the expression of the results of markers like KIM-1 and cytokines in urine samples is required. It seems reasonable to consider the need to correct the results of tubular markers in the urine by urinary creatinine concentration due to a wide variety of factors, which may affect the dilution of urine. Meanwhile, presenting the results of tubular markers in urine such as KIM-1 expressed both as absolute values and values normalized by urinary creatinine seems to be the best strategy. Serum uric acid concentrations were associated with urinary KIM-1 and urinary TNF-alpha, independent of the BMI and eGFR. We hypothesize that serum uric acid at high concentrations, combined with other changes observed in T2D, may contribute to the enhancement of kidney inflammation and tubular damage. However, further longitudinal studies are required to address the real impact of serum uric acid on the development of tubular damage and kidney inflammation in patients with T2D.

## Figures and Tables

**Table 1:** Baseline characteristics and biochemical parameters of the study participants (*n* = 125) stratified according to serum uric acid concentrations.

	Serum uric acid < 6.0 mg/dL	Serum uric acid ≥ 6.0 mg/dL	*P* value
Age (y)	60 (50–68)	59 (46–68)	0.644
Male (%)	23.3	54.3	0.001
BMI (kg/m^2^)	30.2 (26.8–36.0)	34.70 (28.6–40.7)	0.038
Hypertension (%)	69.7	88.6	0.029
Smokers (%)	5.9	16.1	0.082
Diabetes duration (years)	12.0 (8.0–18.0)	10.0 (5.0–21.2)	0.705
Hypoglycemic agents (%)	95.6	91.4	0.359
ACE inhibitors (%)	31.1	61.8	0.002
Other antihypertensive agents (%)	67.0	88.5	0.015
Statin use (%)	74.7	62.9	0.195
Alopurinol use (%)	5.7	6.2	1.000
Insulin use (%)	35.2	20.0	0.132
Serum uric acid (mg/dL)	4.5 ± 0.8	7.0 ± 0.8	<0.001
Fasting glucose (mmol/L)	6.5 (5.5–8.8)	6.6 (5.8–8.4)	0.870
HbA_1c_ (%)	6.9 (6.0–8.5)	6.4 (5.9–8.6)	0.878
HbA_1c_ (mmol/mol)	52 (42–69)	46 (41–70)	0.878
Total cholesterol (mmol/L)	4.6 (4.2–4.9)	4.1 (3.5–5.2)	0.036
LDL cholesterol (mmol/L)	2.6 ± 0.6	2.4 ± 0.8	0.202
HDL cholesterol (mmol/L)	1.2 (1.0–1.5)	1.0 (0.9–1.3)	0.011
eGFR (mL/min/1.73 m^2^)	83.9 ± 21.3	73.5 ± 27.7	0.064
Urinary ACR (mg/g creat)	7.6 (5.2–14.3)	8.0 (5.8–35.3)	0.416

Data are expressed as percentages, mean ± standard deviation, or median and interquartile range. ACE inhibitors: angiotensin-converting enzyme inhibitors; ACR: albumin/creatinine ratio; BMI: body mass index; eGFR: estimated glomerular filtration; HbA_1c_: glycated hemoglobin.

**Table 2:** Biochemical parameters of the study participants (*n* = 125) stratified according to tertiles of serum uric acid concentration.

	1^st^ tertile	2^nd^ tertile	3^rd^ tertile	*P* value
Urinary KIM-1 (pg/mL)	96 (56–144)	77 (58–95)	97 (79–159)	0.039
Urinary KIM-1 (ng/g creat)	121 (76–265)	76 (48–107)	125 (88–200)	0.009
Urinary IL-1 (pg/mL)	93 (73–131)	82 (66–100)	100 (78–143)	0.065
Urinary IL-1 (ng/g creat)	108 (67–192)	88 (53–156)	123 (94–187)	0.057
Urinary IL-6 (pg/mL)	115 (91–144)	99 (92–124)	121 (96–155)	0.098
Urinary IL-6 (ng/g creat)	129 (88–227)	124 (70–184)	171 (103–213)	0.141
Urinary IL-10 (pg/mL)	62 (46–90)	69 (51–135)	64 (39–130)	0.379
Urinary IL-10 (ng/g creat)	76 (47–152)	93 (54–164)	87 (40–178)	0.846
Urinary TNF-alpha (pg/mL)	137 (110–161)	128 (110–139)	132 (106–187)	0.267
Urinary TNF-alpha (ng/g creat)	142 (104–267)	149 (83–189)	176 (124–251)	0.272

Data are expressed as median and interquartile ranges. Parameters are reported as absolute values and as values normalized by urinary creatinine concentration.

**Table 3:** Multiple regression analysis of serum uric acid as a dependent variable adjusting for some clinical and laboratory variables of the study population (*n* = 125).

	*B*	SE_*B*_	*t*	*P* value
*Model 1*				
Male (%)	-0.546	0.357	-1.527	0.133
BMI (kg/m^2^)	0.061	0.017	3.486	0.001
Hypertension (%)	1.806	1.210	1.492	0.142
ACE inhibitors (%)	0.125	0.357	0.349	0.729
Other antihypertensive agents (%)	-1.142	1.176	-0.971	0.336
Total cholesterol (mmol/L)	-0.004	0.203	-0.021	0.984
eGFR (mL/min/1.73 m^2^)	-0.013	0.007	-1.710	0.093
Urinary ACR (mg/g creat)	0.005	0.002	1.977	0.053
Urinary KIM-1 (pg/mL)	-0.001	0.004	-0.176	0.861
Urinary KIM-1 (ng/g creat)	-0.004	0.001	-3.090	0.003
*Model 2*				
Male (%)	-0.304	0.322	-0.944	0.349
BMI (kg/m^2^)	0.055	0.017	3.341	0.001
Hypertension (%)	1.863	1.226	1.519	0.134
ACE inhibitors (%)	0.348	0.318	1.095	0.278
Other antihypertensive agents (%)	-1.234	1.201	-1.027	0.308
Total cholesterol (mmol/L)	-0.175	0.197	-0.890	0.377
eGFR (mL/min/1.73 m^2^)	-0.018	0.006	-2.728	0.008
Urinary ACR (mg/g creat)	0.003	0.002	1.328	0.189
Urinary IL-1 (pg/mL)	-0.025	0.020	-1.254	0.215
Urinary IL-1 (ng/g creat)	0.006	0.013	0.537	0.593
Urinary IL-6 (pg/mL)	-0.021	0.022	-0.991	0.326
Urinary IL-6 (ng/g creat)	0.017	0.011	1.545	0.128
Urinary IL-10 (pg/mL)	0.001	0.006	0.043	0.966
Urinary IL-10 (ng/g creat)	0.002	0.004	0.372	0.711
Urinary TNF-alpha (pg/mL)	0.046	0.017	2.673	0.009
Urinary TNF-alpha (ng/g creat)	-0.024	0.010	-2.464	0.017

Regression coefficients (*B*), standard error of *B* (SE_*B*_), and *t* statistic with corresponding *P* value. Analyses were performed considering urinary concentrations of KIM-1 and cytokines as absolute values and normalized by urinary creatinine.

## Data Availability

The data used to support the findings of this study are included within the article.
